# Inflammation, Immunity, and Cancer

**DOI:** 10.1155/2017/6027305

**Published:** 2017-11-06

**Authors:** Rajesh Singh, Manoj Kumar Mishra, Himanshu Aggarwal

**Affiliations:** ^1^Morehouse School of Medicine, Atlanta, GA, USA; ^2^Alabama State University, Montgomery, AL, USA; ^3^University of Alabama at Birmingham, Birmingham, AL, USA

Inflammation involves interaction between various immune cells, inflammatory cells, chemokines, cytokines, and proinflammatory mediators. It also plays a decisive role at different stages of tumor development, including initiation, promotion, malignant conversion, invasion, and metastasis. In this special issue, we invited few articles to address the issues.

One of the papers of this special issue addresses the role of proinflammatory pathways in the pathogenesis of colitis-associated colorectal cancer. Proinflammatory pathways include several genes that promote tumorigenesis by inducing the production of inflammatory mediators, which provides some promising targets for prevention and therapy. Another paper describes recent advancements in the development of immunotherapies for melanoma, with a specific focus on the identification of neoantigens for the prediction of their elicited immune responses. This paper is expected to provide important insights into the future of immunotherapy for melanoma. In one paper, on patchouli oil, of which an unknown compound identified as patchoulene epoxide (PAO) was found only in the long-stored oil, whose biological activity still remains unknown. Three experimental models were employed to demonstrate that PAO had potent anti-inflammatory activity in vivo. PAO exerted anti-inflammatory activity not only by modulating the pro- and anti-inflammatory cytokines but also by inhibiting iNOS and COX-2 activation via blocking the NF-*κ*B signaling pathway. Based on these results, PAO may serve as a potentially useful therapeutic agent for the treatment of inflammatory diseases, and it is worthwhile to explore other possible pharmacological effects of PAO. Another paper showed the Morita-Baylis-Hillman adducts (MBHA) are a novel group of synthetic molecules that act as anti-inflammatory by modulating inflammatory mediator expression in RAW264.7 macrophages and adenocarcinoma colorectal human HT-29 cells. Another paper discusses osteopontin (OPN) appears to be a key determinant of the crosstalk between cancer cells and the host microenvironment, which in turn modulates immune evasion and describe the role of systemic, tumor-derived and stroma-derived OPN, highlighting its pivotal role at the crossroads of inflammation and tumor progression. Another paper discusses the role of major cytokines and growth factors involved in the endocrine dysfunction of plasma cell disorders. The correction of endocrine dysfunction could thus be beneficial for both the amelioration of cytokine profile and the normalization of hormonal feedback mechanisms. And finally, one of the papers of this special issue is on new treatment approach of hepatocellular carcinoma (HCC). This paper discusses the current status of treating HCC and the effective strategy of oncolytic virus-based immunotherapy for the treatment of HCCs. Oncolytic virotherapy is using oncolytic viruses, which selectively infect and kill cancer cells. In order to enhance immunity against cancer cells, genes elevating oncoimmunity have been engineered within the oncolytic virus. Moreover, a genetically engineered oncolytic virus with mAbs can be generated for advancement treatment in HCC models. Development of biomarker to monitor the treatment outcomes and toxicities would be very helpful in the translational medicinal research.

*Rajesh Singh* *Manoj Kumar Mishra* *Himanshu Aggarwal*

